# Successful Treatment of Complex and Refractory Rosacea Using Platelet‐Rich Plasma Injection Therapy: Case Series and Review of the Literature

**DOI:** 10.1111/jocd.70031

**Published:** 2025-02-09

**Authors:** Xinyue Pang, Lisha Tan, Boyu Zhao, Xiangjun Kong, Huiping Wang, Shuping Hou

**Affiliations:** ^1^ Department of Dermatovenereology Tianjin Medical University General Hospital/ Tianjin Institute of Sexually Transmitted Disease Tianjin China

**Keywords:** case report, platelet‐rich plasma (PRP), rosacea, treatment

## Abstract

**Background:**

Rosacea is a chronic inflammatory skin disease that affects a notable proportion of the adult population. Platelet‐rich plasma (PRP) has been widely used to treat various inflammatory diseases due to its potent anti‐inflammatory and antibacterial properties. Considering its therapeutic potential, PRP is emerging as a promising option for managing rosacea.

**Aims:**

To evaluate the efficacy and safety of PRP therapy in treating distinct clinical presentations of rosacea and explore its potential as an alternative to systemic treatments, particularly in cases where conventional therapies are contraindicated.

**Methods:**

Five patients of rosacea with distinct presentations were treated with PRP therapy. The treatment outcomes were analyzed, and a brief review of relevant literature was conducted to contextualize the findings. Clinical efficacy was assessed using clinical photographs captured at 0 and 30 days after baseline according to the National Rosacea Society Standard (NRSS) grading system. Additionally, the efficacy of combining PRP injections with delicate pulsed light therapy or botulinum toxin injections was assessed.

**Results:**

PRP therapy demonstrated significant efficacy and safety in treating rosacea, offering a viable alternative to systemic treatments. The combination of PRP with delicate pulsed light therapy or botulinum toxin injections showed enhanced therapeutic outcomes, not only alleviating rosacea symptoms but also reducing or eliminating patients' dependence on oral medications.

**Conclusions:**

PRP therapy, both as a standalone treatment and in combination with other modalities, represents a promising approach for managing rosacea. These findings highlight the potential of PRP to address the limitations of conventional therapies and improve patient outcomes. Further research is warranted to validate these results and optimize treatment protocols.

## 1 Introduction

Rosacea is a common, chronic, and recurrent inflammatory facial dermatosis. Rosacea primarily affects individuals over 30 years of age, with documented prevalence rates ranging from 1% to 22% [1]. It is distinguished by paroxysmal flushing, persistent erythema, telangiectasia, papules, and pustules, which significantly impact the activities of daily living and psychological well‐being of those affected. However, a curative therapeutic strategy has yet to be developed [2]. We used platelet‐rich plasma (PRP) injection therapy to treat rosacea, especially in patients who experienced unsatisfactory results from previous treatments.

## 2 Materials and Methods

Five cases of rosacea treated with PRP due to the ineffectiveness of oral medications or contraindications for their use were reported. Informed consents were obtained from all patients (informed consents to discuss their data and informed consents to publish their pictures). This study was carried out on five patients with rosacea, after approval of the research Ethics Committee of Tianjin Medical University General Hospital (approval code IRB2023‐YX‐292‐01).

### 2.1 PRP Preparation

The materials and centrifuge used to prepare PRP were acquired from the Shandong Weigao Group Medical Polymer Co. Ltd., Weihai, China. PRP was prepared by double spin method. An initial centrifugation to separate red blood cells (RBC) is followed by a second centrifugation to concentrate platelets, which are suspended in the smallest final plasma volume [3]. The process begins with whole blood obtained from each patient by peripheral venipuncture, starting with 45 mL of blood drawn from the central cubital vein and mixed with anticoagulant (1:9 ratio). After initial centrifugation at 2000 rpm for 10 min, red blood cells were removed. A second spin at 2200 rpm for 10 min separated the PRP (8 mL) from the remaining blood components. The supernatant and bottom layer of red blood cells were discarded, thus isolating the PRP used for treatment [4].

### 2.2 PRP Injection

Before treatment, baseline photographs were captured, and topical anesthesia was applied. The total amount of PRP is 8 mL, and the injection sites include forehead, bilateral cheeks, and chin, with a space of 0.5 cm between different points of injections. Using a 34G × 1.5 mm needle, PRP (0.02 mL) was injected into the superficial dermis (1.0 ± 0.2 mm deep) at treatment each point. Each patient received one to three sessions of PRP injection therapy at 1‐month interval. After treatment, patients wore a sterile facial mask and an ice compress was applied for 20 min to minimize bruising and mild hemorrhage.

### 2.3 Efficacy Assessment

Clinical efficacy was assessed using clinical photographs captured at 0 and 30 days after baseline according to The National Rosacea Society Standard (NRSS) grading system (0–48) [5], as follows: mild (0–16); moderate (17–32); and severe (33–48).

## 3 Results

**FIGURE 1:**
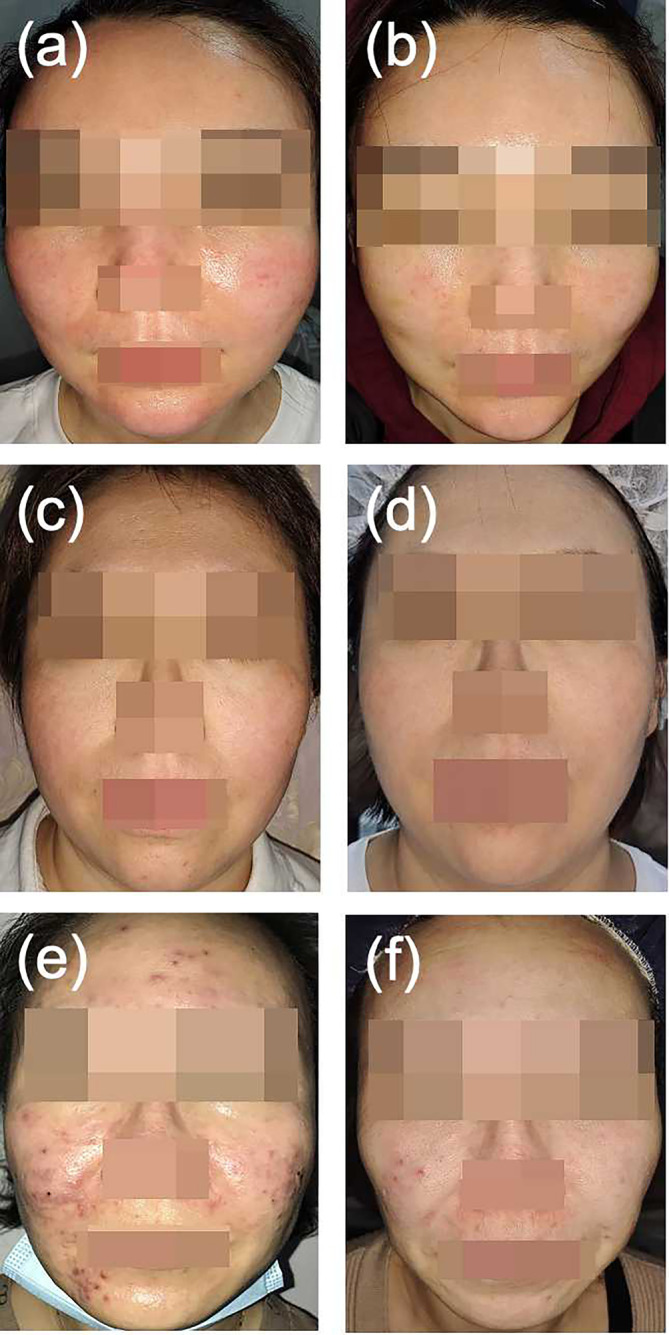
Patient 1: Before PRP injection, facial vascular dilation was found. (b) Patient 1: 1 month after one round of PRP injection, this patient's facial erythema significantly improved. (c) Patient 2: Before treatment, with bilateral cheek vascular dilation. (d) Patient 2: 1 month after three rounds of PRP injections and two sessions of delicate pulsed light (DPL) therapy, this patient showed considerable improvement. (e) Patient 3: Before treatment, with obvious papules and pustules. (f) Patient 3: 6 months after three rounds of PRP injections, the patient's inflammatory lesions exhibited a notable reduction without inducing systemic side effects, and there was no recurrence for 6 months.

**FIGURE 2:**
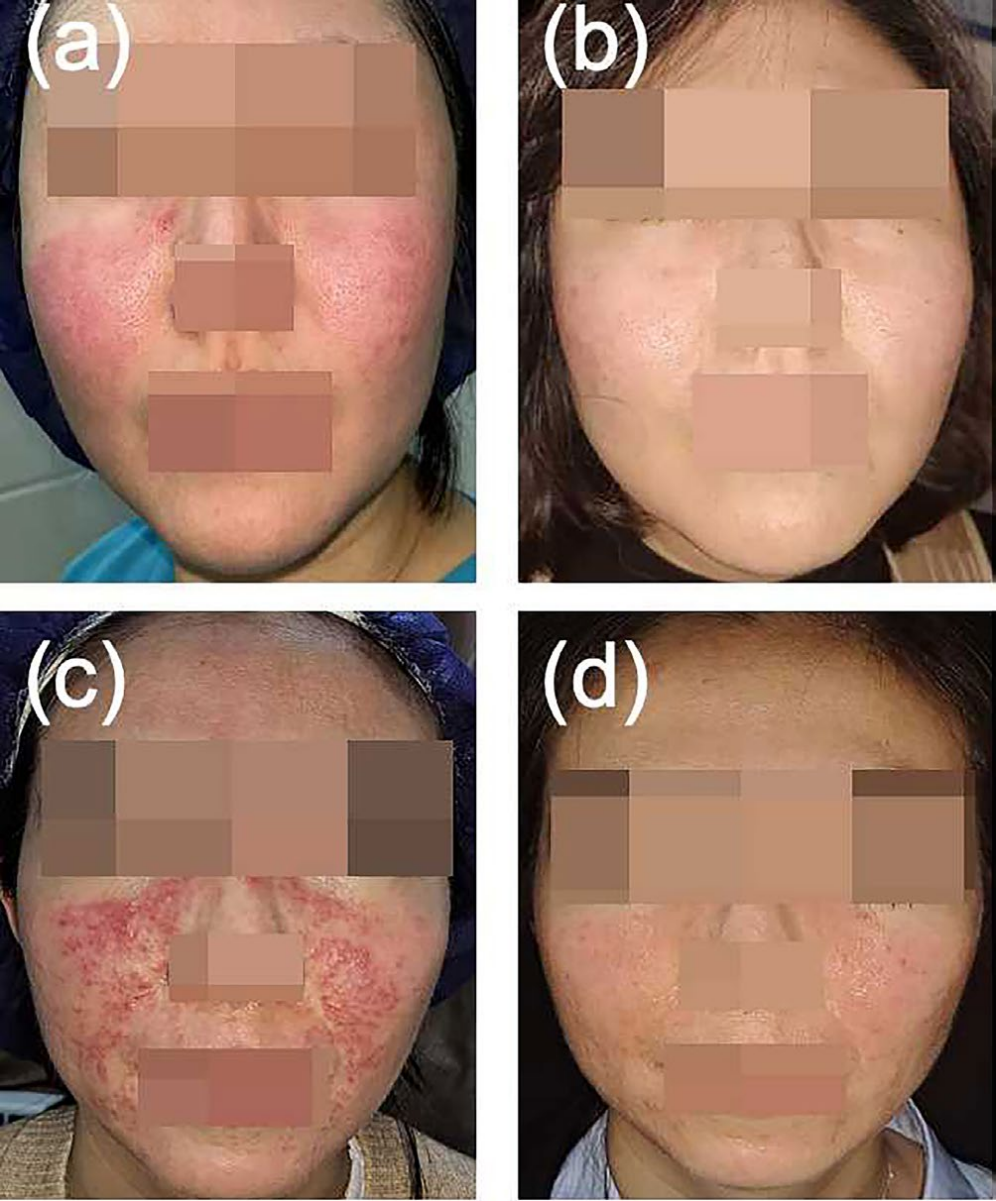
(a) Patient 4: Before treatment, with recurrent facial erythema, papules, and pustules, (b) Patient 4: 1 month after one round of PRP combined with botulinum toxin injection and two rounds of photon therapy. She showed remarkable improvement with no recurrence. (c) Patient 5: Before PRP injection, with distinct facial and perioral erythema, papules, and pustules. (d) Patient 5: 2 months after two rounds of PRP injection. no medication was taken, her rash significantly improved and did not recur.

After PRP injections, telangiectasia, erythema, and papules were relieved in patients compared with pretreatment photographs (Figures 1 and 2). Based on the grading system described above, clinical rosacea scores for all patients demonstrated a decrease at Day 30 compared with baseline. Particularly noteworthy were Patients 3 and 5, in whom scores decreased substantially, resulting in a reduction in grading from moderate to mild, reflecting a highly positive response to treatment (Table 1).

**TABLE 1:** NRSS grading with clinical scores of each patient.

Day (study status)	NRSS grading with clinical scores of patients				
	Patent 1	Patent 2	Patent 3	Patent 4	Patent 5
Day 0 (baseline)	Mild (10)	Mild (7)	Moderate (18)	Mild (13)	Moderate (20)
Day 30 (after treatment)	Mild (5)	Mild (4)	Mild (10)	Mild (5)	Mild (11)

Patient 1, a 48‐year‐old woman with a two‐year history of erythematotelangiectatic rosacea, was treated with minocycline hydrochloride. The papules subsided, leaving facial vascular dilation. The patient's facial erythema significantly improved after two rounds of PRP injections, which is consistent with the reported efficacy of PRP in improving microvascular circulation and reducing erythema (Figure 1a,b).

Patient 2 was a 35‐year‐old woman with a 2‐year history of persistent bilateral cheek vascular dilation and diagnosed with erythematotelangiectatic rosacea. Despite previous treatment attempts, the condition remained unimproved. The patient underwent a comprehensive therapeutic approach involving three rounds of PRP injections strategically combined with two sessions of delicate pulsed light (DPL) therapy (Harmony XL, Alma Lasers Ltd., Caesarea, Israel) at a wavelength of 500 nm, a pulse duration of 12 ms, and fluence of 6–7 J/cm^2^. The patient exhibited considerable improvement after therapy. The combination of PRP and photon rejuvenation enhanced skin rejuvenation and vascular reduction (Figure 1c,d).

Patient 3 was a 49‐year‐old woman with a one‐year history of facial erythema, papules, and pustules and was diagnosed with papulopustular rosacea for which she refused medication due to renal insufficiency. A facial injection of PRP was administered without any oral medication. After three rounds of treatment, the patient's skin lesions improved significantly, with no recurrence for 6 months. This case demonstrated the role of PRP in reducing inflammatory lesions without the use of systemic medications (Figure 1e,f).

Patient 4, a 36‐year‐old woman diagnosed with papulopustular rosacea, experienced a year‐long battle with recurrent facial erythema, papules, and pustules necessitating long‐term oral methylprednisolone. However, her condition remained challenging, with each reduction in methylprednisolone dosage triggering a relapse of rash, reflecting the refractory nature of her condition. Seeking an alternative solution, the patient underwent a treatment regimen comprising a single round of PRP injection combined with botulinum toxin therapy and two rounds of DPL (Harmony XL) at a wavelength of 500 nm, a pulse duration of 12 ms, and fluence of 6–7 J/cm^2^. Remarkably, this innovative combination therapy achieved significant improvement, facilitating a gradual reduction in methylprednisolone dosage until eventual discontinuation. This result suggests a synergistic effect of PRP and botulinum toxin in the treatment of rosacea (Figure 2a,b).

Patient 5, a 32‐year‐old woman with a 2‐year history of papulopustular rosacea, relied on long‐term oral administration of minocycline hydrochloride and hydroxychloroquine sulfate to manage her condition. Her symptoms recurred every time she discontinued medication, underscoring the refractory nature of her rosacea. Facial inspection revealed facial and perioral erythema, papules, and pustules. In seeking alternative treatment options, the patient opted for PRP injections and, after undergoing two rounds, her rash significantly improved and she discontinued oral medications. This case highlights the potential of PRP in the management of refractory cases of rosacea (Figure 2c,d).

## 4 Discussion

Current treatment methods for rosacea include topical medications, including metronidazole, azelaic acid, clindamycin, erythromycin, benzoyl peroxide, pimecrolimus, tacrolimus ointment, and brimonidine, as well as systemic treatments, such as doxycycline, minocycline, hydroxychloroquine, isotretinoin, and carvedilol. Advanced therapeutic modalities for persistent facial telangiectasia include pulsed dye laser (PDL 585/595 nm), intense pulsed light (IPL 500–1200 nm), potassium titanyl phosphate (KTP) laser, and long‐pulse, neodymium‐doped yttrium aluminum garnet (Nd:YAG) laser treatments [6]. Despite myriad options, the treatment of rosacea remains challenging.

In recent years, PRP has emerged as a novel biological treatment modality, which has made significant strides in the field of medical esthetics. In addition to its use in skin rejuvenation, autologous fat grafting, scar reduction, hair loss, and pigmentary disorders, PRP has also demonstrated efficacy in treating rosacea [7]. Clinical treatment of rosacea with PRP alone has yielded good results [4], suggesting that PRP may be a viable option.

However, the exact mechanism of action of PRP in the treatment of rosacea remains unclear. Studies have shown that upon injection into the interstitial spaces of the skin, PRP is rapidly activated, releasing various growth factors, including epidermal growth factor and transforming growth factor‐beta, which promote cell proliferation and matrix production, thereby accelerating repair of skin lesions [8]. Additionally, PRP has both bactericidal and antibacterial effects [9]. The anti‐inflammatory effect of PRP appears to be induced by the suppression of cyclooxygenase and production of prostaglandins, and by increasing the intracellular expression of inflammation‐controlling cytokines [10].

Positive outcomes of PRP injection in the treatment of rosacea were observed in all five cases described. Moreover, a combination of PRP injections with DPL therapy or botulinum toxin injections demonstrated significant efficacy in the treatment of this condition. This combination therapy not only alleviated the symptoms of rosacea but also helped patients reduce or eliminate their dependence on oral medications. This emphasizes the need for personalized and multidisciplinary approaches to rosacea management, including the exploration of alternative therapies and the development of tailored treatment plans to address the individual needs of patients with this chronic skin disease. Moreover, this therapeutic method is relatively safe and offers a gentler and more sustainable treatment option for patients with rosacea. However, there are certain limitations in this study: The small sample size and lack of male patients may affect the stability and representativeness of the results. Additionally, in Patient 2, PRP was combined with DPL, and in Patient 4, PRP was combined with botulinum toxin and DPL, so the improvement may not be entirely attributed to PRP. Further studies are required on large number of patients, including male participants, and the establishment of a control group with longer period of follow‐up to scientifically assess the clinical efficacy of PRP.

## Author Contributions

**Xinyue Pang:** writing – review and editing, writing – original draft, methodology, investigation, formal analysis, data curation, conceptualization. **Lisha Tan:** writing – review and editing, writing – original draft, conceptualization. **Boyu Zhao:** writing – review and editing, formal analysis, adta curation, conceptualization. **Xiangjun Kong:** writing – review and editing, methodology, conceptualization. **Huiping Wang:** supervision, project administration, funding acquisition, conceptualization. **Shuping Hou:** writing – original draft, writing – review and editing, visualization, validation, supervision, project administration, funding acquisition, conceptualization.

## Ethics Statement

The patients in this manuscript have given written informed consent to publication of their case details.

## Conflicts of Interest

The authors declare no conflicts of interest.

## Data Availability

The data underlying this article will be shared on reasonable request to the corresponding author.
